# Polymer Composites with 0.98 Transparencies and Small Optical Energy Band Gap Using a Promising Green Methodology: Structural and Optical Properties

**DOI:** 10.3390/polym13101648

**Published:** 2021-05-19

**Authors:** Muaffaq M. Nofal, Shujahadeen B. Aziz, Jihad M. Hadi, Wrya O. Karim, Elham M. A. Dannoun, Ahang M. Hussein, Sarkawt A. Hussen

**Affiliations:** 1 Department of Mathematics and General Sciences, Prince Sultan University, P.O. Box 66833, Riyadh 11586, Saudi Arabia; muaffaqnofal@gmail.com; 2 Hameed Majid Advanced Polymeric Materials Research Laboratory, Physics Department, College of Science, University of Sulaimani, Qlyasan Street, Sulaimani 46001, Kurdistan Regional Government, Iraq; ahang.hussein@univsul.edu.iq (A.M.H.); sarkawt.hussen@univsul.edu.iq (S.A.H.); 3 Department of Civil Engineering, College of Engineering, Komar University of Science and Technology, Sulaimani 46001, Kurdistan Regional Government, Iraq; 4 Department of Medical Laboratory of Science, College of Health Sciences, University of Human Development, Sulaimani 46001, Kurdistan Regional Government, Iraq; jihad.chemist@gmail.com; 5 Chemistry Department, College of Science, University of Sulaimani, Qlyasan Street, Sulaimani 46001, Kurdistan Regional Government, Iraq; wrya.karim@univsul.edu.iq; 6 Associate Director of General Science Department, Woman Campus, Prince Sultan University, P.O. Box 66833, Riyadh 11586, Saudi Arabia; elhamdannoun1977@gmail.com

**Keywords:** metal complex, small bandgap PVA composites, XRD and FTIR, UV-vis study, localized density of state, bandgap study

## Abstract

In this work, a green approach was implemented to prepare polymer composites using polyvinyl alcohol polymer and the extract of black tea leaves (polyphenols) in a complex form with Co^2+^ ions. A range of techniques was used to characterize the Co^2+^ complex and polymer composite, such as Ultraviolet–visible (UV-Visible) spectroscopy, Fourier transform infrared spectroscopy (FTIR), and X-ray diffraction (XRD). The optical parameters of absorption edge, refractive index (*n*), dielectric properties including real and imaginary parts (*ε_r_*, and *ε_i_*) were also investigated. The FRIR and XRD spectra were used to examine the compatibility between the PVA polymer and Co^2+^-polyphenol complex. The extent of interaction was evidenced from the shifts and change in the intensity of the peaks. The relatively wide amorphous phase in PVA polymer increased upon insertion of the Co^2+^-polyphenol complex. The amorphous character of the Co^2+^ complex was emphasized with the appearance of a hump in the XRD pattern. From UV-Visible spectroscopy, the optical properties, such as absorption edge, refractive index (*n*), (*ε_r_*), (*ε_i_*), and bandgap energy (*E_g_*) of parent PVA and composite films were specified. The *E_g_* of PVA was lowered from 5.8 to 1.82 eV upon addition of 45 mL of Co^2+^-polyphenol complex. The N/m* was calculated from the optical dielectric function. Ultimately, various types of electronic transitions within the polymer composites were specified using Tauc’s method. The direct bandgap (DBG) treatment of polymer composites with a developed amorphous phase is fundamental for commercialization in optoelectronic devices.

## 1. Introduction

The utilization of polymer materials instead of metallic ones has become more widespread in recent years [1]. This is due to the structural characteristics of the former, such as the tunability of optical bandgaps [2]. Polymer composites with enhanced optical properties have been utilized in various applications, such as optical networking and sensors, light-emitting diodes, solar panels, data storage systems, polarizers, and even biomedical applications. Polymers, especially inorganic, nanocomposite ones, can be fabricated by incorporating inorganic nanoparticles that are then used in optoelectronic systems [3], making it possible manipulate their optical properties [4].

This modification in optical properties by increasing absorption intensity is caused by electrostatic interactions between the nanofillers and the host polymers [5]. It is believed that nanofillers introduce localized charge carrier levels within the polymer matrices as trapping sites. Thus, it is now possible to accurately measure the optical constant. Pollution caused by heavy metals is one of the most significant environmental concerns today [6]. Heavy metals are defined as metals and metalloids with an atomic density greater than 4000 kg m^−3^; in other words, five times greater than that of water [7]. In principle, sorption is a mass transfer process through which a substance transports from the liquid phase to the solid surface. The solid surface on a substance of interest is bound by physical and/or chemical interactions [6]. One well-known method of removing toxic heavy metals is precipitation. Accordingly, the solubility difference of metal precipitates can be exploited for separating by adding suitable selective anions [8]. This traditional means of removal of heavy metals is not free of drawbacks; for instance, considerable surface land contamination occurs, and a sludge dewatering facility, skillful operators, and a multiple basin design are required. Several methodologies are available for heavy metal removal from wastewater, such as biosorption, neutralization, precipitation, ion exchange, adsorption, biosorption, neutralization, precipitation, ion exchange, and adsorption [9]. The straightforward and easy way involves the use of a selective precipitating agent. Several appropriate bio sorbents, such as potato peels, sawdust, black gram husk, eggshell, seed shells, coffee husks, sugar-beet pectin gels, or citrus peels, have been studied in this respect [10]. Plant materials have shown their potential in nanoparticle synthesis, especially in the biosynthesis process, acting as both reducing and capping agents. Moreover, almost all parts of the plants can be utilized for nanoparticle synthesis, including the leaves, flowers, seeds, stems, fruits, latex, and calli. Furthermore, dead and dried plants can also be used for the synthesis of nanoparticles [11].

Photonic materials that are highly efficient and photostable can also be helpful in biophotonic applications, such as dye-doped polymers. Unique applications of these materials include optical microscopy and nanoscopy, as these methods provide relatively long observation times and improved spatial resolution [12]. For example, duvenhage-PMMA is doped with Alq3 for utilization in optoelectronics.

Polyvinyl alcohol PVA is a water-soluble crystalline polymer that is nontoxic and biocompatible. It is therefore cheap and readily degradable. It has excellent chemical and mechanical stability, making ideal for applications such as electrochemical and optoelectronic devices. Also, due to its polarity and hydrophilic properties, PVA can interact with a variety of organic and inorganic materials [13,14,15,16,17]. It is worth noting that the degradation of organometallic and conjugated polymers is fast, resulting in reduced device performance and efficiency [18]. Thus, it is of great importance to modify PVA by narrowing the bandgap polymer by metal complex incorporation. This opens a new perspective for research in optical materials and developing optoelectronic and photonic devices.

A remarkable drop in optical bandgap was observed in our previous work regarding polymer a composite based on the aluminum (III) metal polyphenol complex in a PVA polymer [19]. Also, adding Co^2+^-polyphenol metal into polymer matrices to enhance their optical properties represented novel research.

In materials science, to change magnetic, transport optical, dielectric, and structural properties, the focus is typically on molecular charge-transfer materials. One can determine the optical, electrical, and photoelectrical properties from the charge transfer mechanism in complexes, as is required in many electro-physical and optical processes [20].

Herein, black tea extract solutions are used to sustainably synthesize a Co^2+^-polyphenol complex. These solutions are enriched with polyphenols that interact strongly with the Co^2+^ ion to produce the Co^2+^-polyphenol complex. The main constituents of tea leaves, such as caffeine and polyphenols, were documented by Zielinski et al. [21].

It should be noted that the coordination chemistry has complex coordinated, complex compounds or only complexes. Coordination compounds are recognized as the lights and empty orbital metallic centers coordinated by donors of electron pairs [22].

It has previously been documented that electrical double-layer capacitor (EDLC) devices can be modified via the incorporation of a chitosan-based polymer electrolyte of the Zn^2+^-polyphenol complex, yielding improvements in performance [23,24].

In this work, we incorporated a Co^2+^-polyphenol complex into a PVA polymer in order to achieve the desired optical properties. In this report, it is shown that the amorphous phase of PVA composites can be increased and the optical band gap energy (*E_g_*) reduced. The green methodology and desired optical properties, notably, the small *E_g_* of the polymer composite, indicate the significance of the present study.

## 2. Materials and Methods

### 2.1. Materials

Two raw materials were purchased from Sigma-Aldrich (Kuala Lumpur, Malaysia), and used without modification: polyvinyl alcohol (PVA) powder (99% purity) with an average molecular weight of (85,000 to 124,000) and cobalt (II) nitrate hexahydrate [Co (NO_3_)_2_]·6H_2_O (99.99% purity) (291.03 g/mol). Black tea leaves were acquired from the market.

### 2.2. Metal Complex Synthesis and Composite Fabrication

In the extraction process, distilled water was used as a solvent for extracting the tea leaf components; 50 g of black tea leaf was added to 250 mL distilled water, maintained at a temperature at 90 °C and kept away from sunlight. The extraction process of the black tea leaves lasted 10 min. Then, filtration was carried out using Whatman filter paper (Whatman 41, cat. no. 1441) with an average pore radius of 20 µm. In a separate solution, 10 g of cobalt (II) nitrate hexahydrate [Co (NO_3_)_2_]·6H_2_O was dissolved in 200 mL of distilled water. Both solutions were mixed at 80 °C and then stirred for around 10 min to produce a Co^2+^-polyphenols complex.

Finally, the Co^2+^-polyphenol complex solution was identified from the physical appearance of a green precipitate in the form of a turbid solution that settled out from the solution of the dark-colored tea leaves. The solution was allowed to cool to room temperature, and then then washed using distilled water several times. To disperse the synthesized Co^2+^-polyphenol complex materials, 100 mL of distilled water was used.

The composite samples were prepared from PVA and the Co^2+^-polyphenol complex as raw materials using the casting method. In this methodology, 1 g of PVA powder was added to 40 mL of distilled water; the mixture was then stirred for 60 min using a magnetic stirrer, and maintained at a temperature of around 80 °C. Afterward, this solution was left to cool to room temperature. In the incorporation process, two portions of Co^2+^-polyphenol complexes were added to the PVA homogenous solution in steps of 30 mL from 0–60 mL solution. To homogenize these solutions, stirring was conducted for around 50 min. The samples were labelled as SPMC_0, SPMC_1, and SPMC_2, corresponding to PVA portions with 0, 30, and 60 mL of Co^2+^-polyphenol complexes. The casting process was performed by adding these solutions to a series of Petri dishes and leaving them at room temperature. To make the films dried, and before characterizations were performed, a desiccator containing blue silica gel was used. The thickness of pure PVA and composite films was estimated to be between 0.012 and 0.015 cm.

### 2.3. X-ray Diffraction

The X-ray diffraction patterns for the pure PVA and composite films were obtained at room temperature, using a Bruker AXS with a working voltage and current of 40 kV and 45 mA, respectively. The monochromic beam of X-ray radiation was set at 1.5406 Å, with glancing angles (2θ) between 10° and 80° and a step size of 0.05°.

### 2.4. Fourier Transform Infrared Spectroscopy (FTIR)

To determine the nature of the interaction between the composite components, an FTIR spectrophotometer was used in the range 400–4000 cm^−1^ at a resolution of 2 cm^−1^.

### 2.5. UV-Visible Spectroscopy

The UV-Visible absorption spectra of the composite films were recorded using a Jasco V-570 UV-Visible spectrophotometer.

## 3. Result and Discussion

### 3.1. UV-Vis Study of Co^2+^-Polyphenol Complex

**Figure 1:**
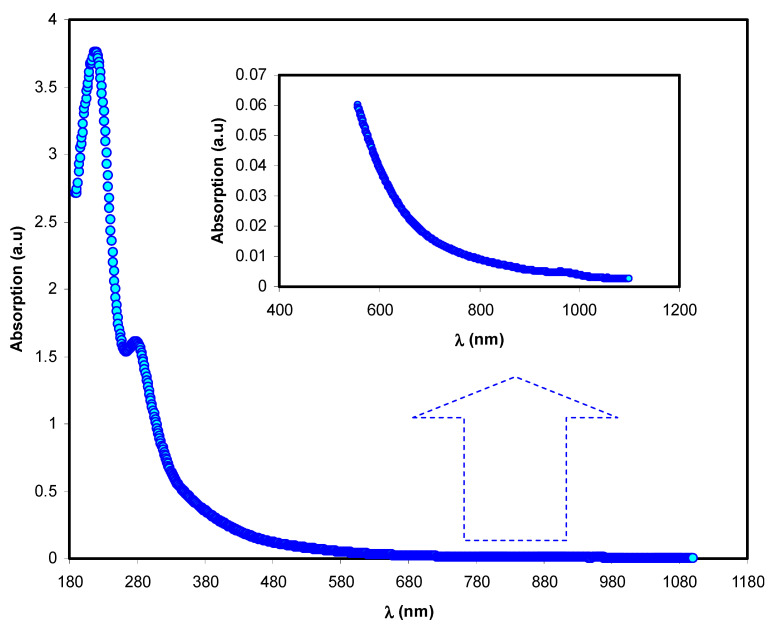
Absorption spectra versus wavelength for the Co^2+^-polyphenol complexes.

The absorption spectrum of the Co^2+^-polyphenol complex colloidal suspension solution is shown in Figure 1. The absorption spectrum occupies the entire visible range, which begins in the visible range and ends in the UV range. The main features of the absorption spectrum of organometallic-based materials and semiconductors are well-known [25]. Wang et al. [26] documented Fe^2+^-polyphenol complexes from extractions of rosemarinus officinalis, eucalyptus tereticornis, and melaleuca, and found that the UV-Vis spectrum for the Co^2+^-polyphenol complex was in agreement with that of the Fe^2+^-polyphenol complex.

According to the spectrum observed at 200 and 350 nm, the reaction in this range can be attributed the electronic transition of n–π* in methylxanthines and catechins, which are composed of theophylline, theobromine, and caffeine. The band absorption at ~278 nm correlates to the C=O chromophore in caffeine [27,28,29]. Metal nanoparticles are mainly characterized by absorption of the surface plasmon resonance (SPR) in the UV-Visible range [30]. It is interesting to note that no sign of SPR absorption of the Co^2+^-polyphenol complex is seen (Figure 1). This may be attributable to the absence of metal characteristics in the Co^2+^-polyphenol complex due to the polyphenol capping phenomenon. However, an SPR peak for chitosan-based polymer electrolytes was recorded between 500 to 800 nm, due to the presence of copper nanoparticles [31].

### 3.2. FTIR Study of Black Tea and Co^2+^-Polyphenol Complex

**Figure 2:**
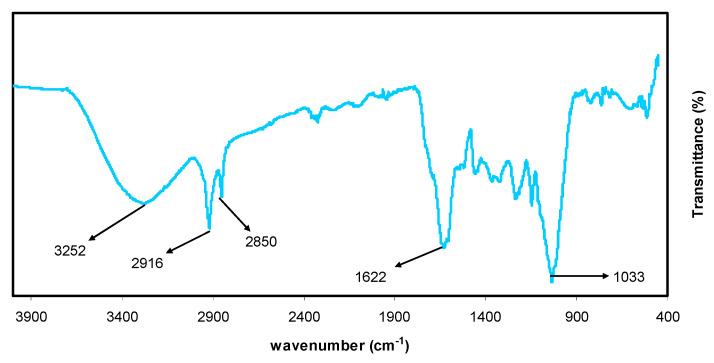
FTIR spectra for black tea leaf extract.

**Figure 3:**
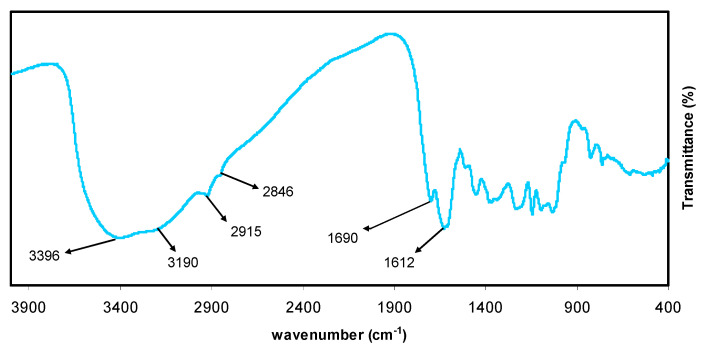
FTIR spectrum of colloidal Co (II)-complex.

Figure 2 presents the FTIR spectra for the black tea extract. The appearance of multiple peaks is the main characteristic of the FTIR spectrum. Peaks in the range of 2916–2851 cm^−1^ may be attributed to the C-H stretching of aliphatic groups and carboxylic acid [32,33]. Also, a band at 1623 cm^−1^ was assigned to C=C stretching in the aromatic ring. [32,34]. It is worth noting that the whole FTIR spectrum agrees well with those obtained in previous studies [33,35]. It was recently discovered that the caffeine spectrum includes many variations in the region of 1700–400 cm^−1^ (see Figure 3). These modifications indicate the presence of a variety of functional groups that have binding and stretching motions, such as methyl, carbonyl, pyrimidine fragments, and imidazole [36,37]. According to the FTIR spectra, carboxylic acid, polyphenol, and amino acids are the main functional groups in tea. It has been reported in the literature that polyphenols can interact strongly with metal cations, particularly cobalt, forming colloidal Co^2+^ polyphenol complex suspensions [38].

The FTIR spectrum of the Co^2+^-polyphenol complex is shown in Figure 3. Figure 2 and Figure 3 show a series of peaks in the region of 1700–400 cm^−1^, the intensities of which were typically altered.

This work mainly focuses on Co^2+^ ion colloidal as one of the properties of the Co^2+^ polyphenol complex using FTIR. Wang et al. [39] studied an extract of eucalyptus leaves in the synthesis of Fe-polyphenols complex. It was noted that the complex was formed via interaction between Fe^2+^ and polyphenols.

The mixture consists of a green solution (filtrate) and colloidal suspension at the top and the bottom, respectively, confirming the formation of the Co^2+^-polyphenol complex. The characteristic bands of black tea are repeated in the FTIR spectrum of Co^2+^-polyphenol complexes; however, the peak intensities decreased (see Figure 3).

Interestingly, the bands at 2916 and 2851 cm^−1^ in black tea shifted when the Co^2+^ polyphenol complexes formed, instead appearing at 2913 and 2844 cm^−1^, respectively. This can be explained by the formation of coordination bonds between Co^2+^ ions and polyphenols where vibrational reduction occurs, leading to an increase in reducing mass. The coordination bond formation between and polyphenols results from the attraction between the ligand pairs and empty orbitals in the Co^2+^ ions [21]. The coordination mechanism between Co^2+^ ion and the ligands of interest will be schematically depicted in the next section. Wang et al. [26] documented the synthesis and characterization of iron-polyphenol complexes using various extracts, including eucalyptus tereticornis, melaleuca nesophila, and rosemarinus. The authors showed that iron ions react with polyphenols, leading to the formation of iron-polyphenol complexes. ÓCoinceanainn et al. reported the complexation of aluminum (III) with theaflavin using FTIR. The polyphenolic compounds are ligands found in the extracts of black tea [40].

**Figure 4:**
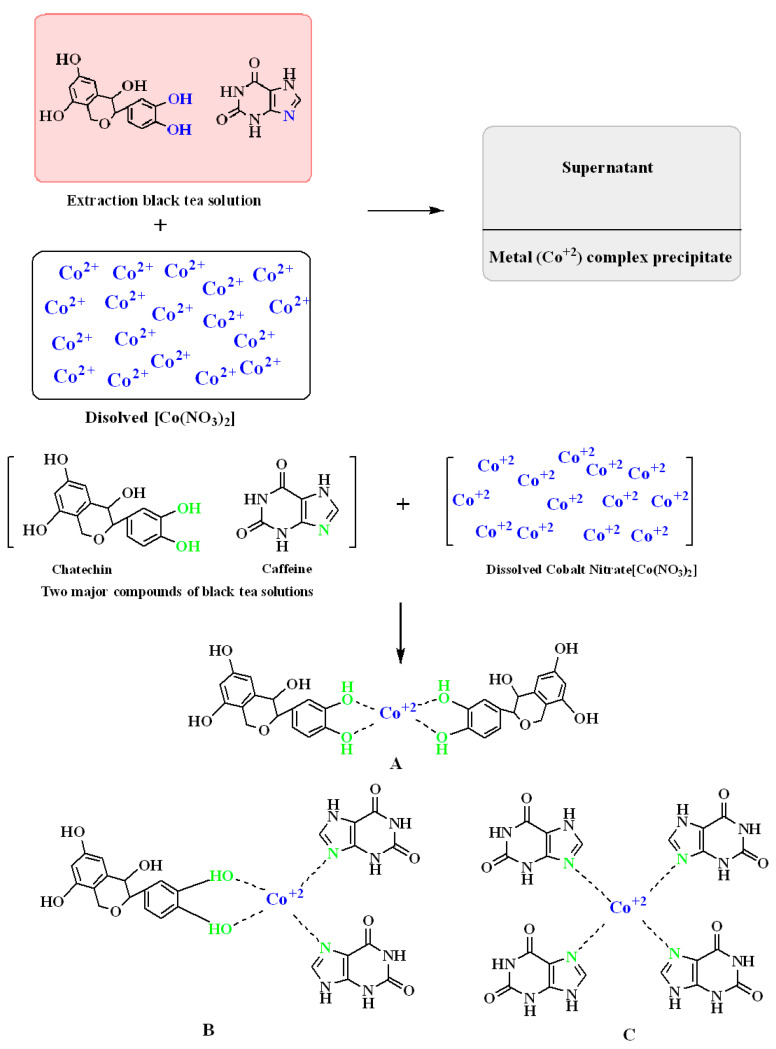
Proposed structure for the mechanism of the formation of Co^2+^-polyphenol complexes.

The nature of the interaction between Co^2+^ ions and caffeine and polyphenols of tea extracts can be assessed using FTIR analysis, as shown in Figure 4. The chemistry of interaction between Co^2+^ ion and tea extracts comprises several complexes (see Figure 4). These interactions between metal ions and tea constituents have been confirmed previously [20,41,42,43]. Three possible complexes are shown in Figure 4. As usual, the Co^2+^-polyphenol complex is strongly anticipated (see Figure 4A), as is that of Co^2+^- caffeine (Figure 4C).

Furthermore, as shown in Figure 4B, there is a possibility of interactions between Co^2+^ and polyphenol and caffeine in a complex. Previously, the EPR method has been used to investigate the formation of complexes by metal ions and polyphenols of extracts of black tea [41]. In the current study, the nature of metal complex formation was studied using FTIR.

### 3.3. XRD and FTIR Study of PVA Based Composites

**Figure 5:**
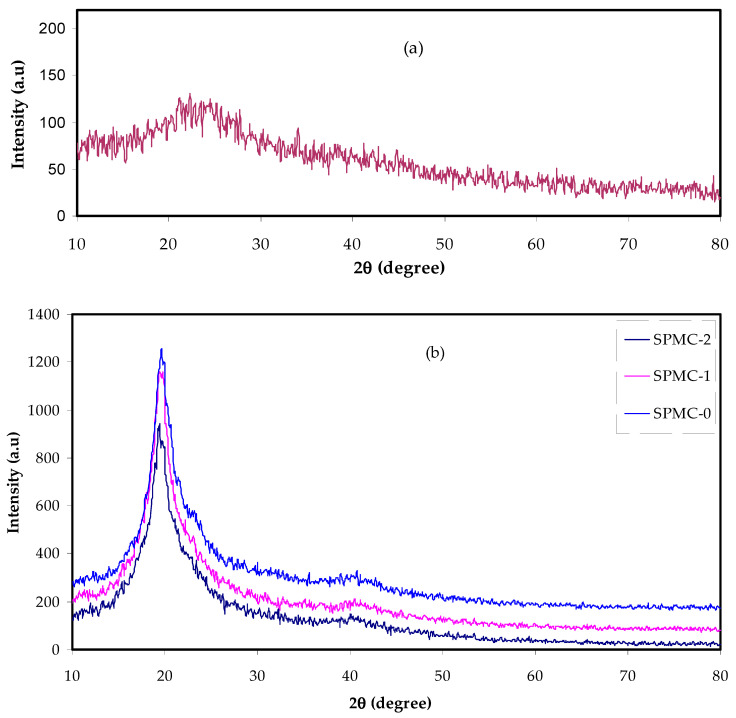
XRD pattern for (**a**) Co^2+^-polyphenol complex and (**b**) neat PVA and composite samples.

The XRD patterns of Co^2+^-polyphenol complex, neat PVA and PVA composite films are shown in Figure 5a,b. It is seen (Figure 5a) that the structure of the synthesized Co^2+^-polyphenol complex is mainly amorphous, as evidenced by the absence of crystalline peaks over the 2θ degree range. The hump which appeared in the XRD pattern of Co^2+^-polyphenol complex is characteristic of amorphous materials. On the other hand, two peaks can be distinguished in the XRD pattern of neat PVA and their composites (see Figure 5b). The two peaks lie at 2θ = 20° and 40° in the XRD pattern of neat PVA, and are separated completely, correlating with crystalline phase [44,45]. Despite the intense decrease of these peaks, in the doped PVA spectra, we observed the appearance of one and disappearance and broadening of another, especially at 2θ = 40°. These changes in intensity and broadening of the peak, in particular that at 2θ = 20°, designates amorphous phase development within the PVA matrix [46,47,48]. It was previously demonstrated that the addition of Zn^2+^-polyphenol complex into a chitosan polymer-based electrolyte significantly extended the amorphous area [17].

At this stage, it was of crucial importance to study the XRD spectra in an attempt to distinguish between the amorphous and crystalline peaks [49]. It is intuitive that broadened peaks are characteristic of the amorphous phase; on the other hand, a narrow (or sharp) peak is a feature of the crystalline phase. It can be seen that the crystalline peaks in SPMC 1 and SPMC 2 declined and broadened as the concentration of Co^2+^-polyphenol complex increased. As seen in SPMC 2, adding 60 mL of Co^2+^-polyphenol complex induced a major difference in peak form, resulting in decreasing intensity of the crystalline peak (see Figure 5b).

The main features of the present XRD pattern are in good agreement with those previously recorded [50,51]. Wang et al. [26] used various extracts to fabricate Fe-polyphenol complexes, such as eucalyptus tereticornis, melaleuca nesophila, and rosemarinus officinalis. The authors confirmed the presence of the amorphous phase of the Fe- polyphenol complexes from the XRD spectra, in which no prominent diffraction peaks were seen for the three plants.

**Figure 6:**
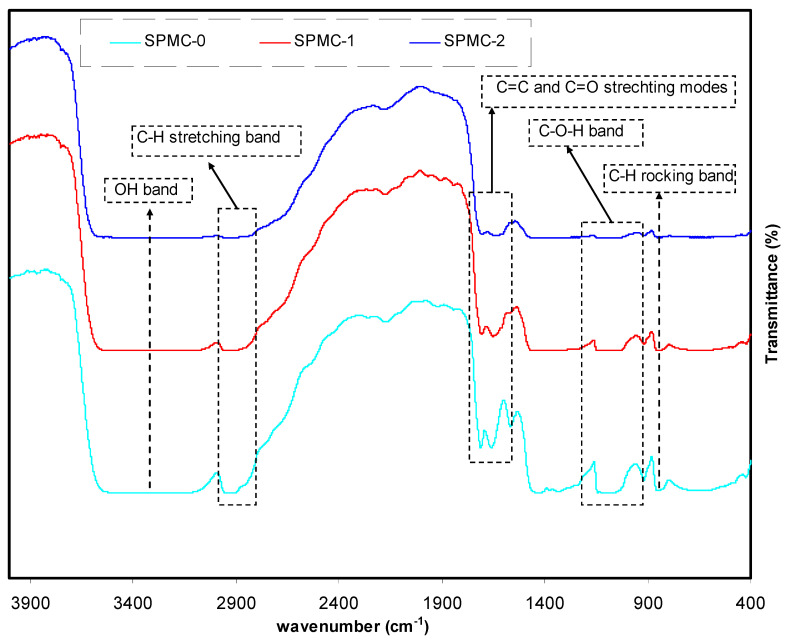
FTIR spectra of the SPMC_0, SPMC_1, and SPMC_2 in the region of 400–4000 cm^−1^.

The FTIR spectra of parent PVA and PVA doped with Co^2+^-polyphenol complex are shown in Figure 6. As shown, the absorption peak at 824 cm^−1^ is caused by C-H rocking of pure PVA [44]. The spectra of doped samples show both a shift and a decrease of the peak position and intensity. In pure PVA, -CH_2_ wagging yields an absorption peak at 1410 cm^−1^ [52]. In the spectrum of pure PVA, a band at 2908 cm^−1^ occurred, due to the presence of C-H asymmetric stretching vibrations [53,54]. In the spectra of doped samples, the peak changes position and becomes faint, respectively. Two types of interactions can be taken into consideration: the reaction between the Co^2+^-polyphenol complex colloidal and PVA functional groups, and the adsorption of the Co^2+^-polyphenol complex colloidal on the PVA functional groups. The vibrational modes of functional groups become weak as a consequence of the formation of bulky molecular systems. [46]. Table 1 presents various vibrational modes.

### 3.4. Optical Properties of PVA Composites

#### 3.4.1. Transmittance and Absorbance Analysis

**Figure 7:**
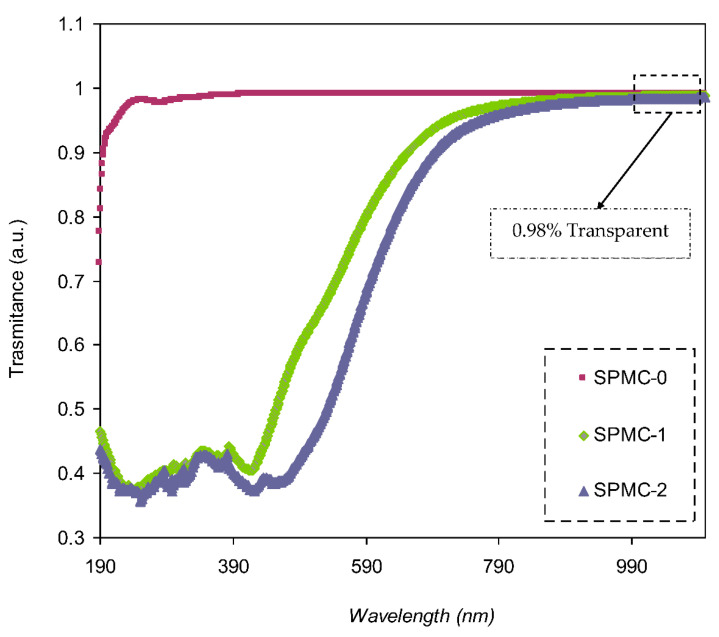
Absorption spectra of parent PVA (SPMC_0) and PVA composites absorption spectra.

The transmittance of parent and doped PVA with various quantities of Co^2+^-polyphenol complex is presented in Figure 7. Pure PVA possesses relatively high transparency beyond the visible region, i.e., over 98%, whereas below the visible region, the transparency drops due to the high absorption of the films. PVA doped with 30 and 60 mL Co^2+^-polyphenol complex exhibited lower transparency, reaching almost 45% at the UV region, while transparency increased to 0.98% in the visible region. This may be attributed to the scattering and relatively high refractive index of highly doped films at lower λ (nm). It was noted that the spectrum possessed a shoulder in the parent PVA (UV region); on the other side, this shoulder did not exist. This indicates strong interactions between the functional groups within the polymer chains and the added Co^2+^-polyphenol complex, causing a drop in the transparency of the doped films. Molkenova et al. [55] reported on Europium metal thin films doped with titanium oxide (TiO_2_). Their findings revealed a transmittance percent of 83.3% for Eu doped film in the visible range. Also, the transparency value for the blend polymers of poly (propylene carbonate) PPC and poly (ethyl cyanoacrylate) PECA with caffeic acid was found to be around 80%, as documented by Quilez-Molina et al. [56].

**Figure 8:**
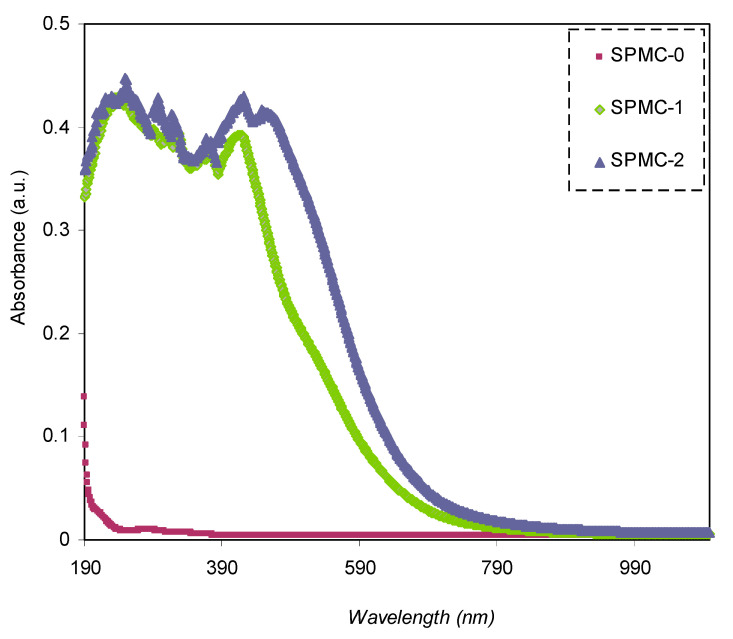
Absorption spectra of parent PVA (SPMC_0) and PVA composites absorption spectra.

The absorption spectra of both parent PVA and doped PVA are shown in Figure 8. It may be seen that the responses of the absorption spectra of the PVA doped samples cover almost all UV-visible regions, extending to the near-infrared range. Pure PVA has no absorption response in the visible to near-infrared range because of the absence of free electrons, causing it to be almost transparent. Furthermore, the relatively high-energy (UV region) photons result in electron transport across the bandgap between the valence and conduction bands.

From both the absorption spectra of pure PVA and doped PVA samples, it is seen that composite samples containing Co^2+^-polyphenol complex display extreme absorption throughout the entire UV-Visible range. It may also be seen that as the content of the Co^2+^-polyphenol complex increased in the PVA, and the absorption band position shifted to a greater wavelength (i.e., lesser energy). It is vital technologically that the synthesized polymer composites have the desired optical characteristics for applications in various industries, for instance, photonics, solar cells, and optoelectronic instruments [57]. To achieve high-power conversion efficiency in solar cells, the light-harvesting potential needs to be manipulated via the absorption band widening and shifting the position of absorption bands to near IR region responses, which, in turn, increase the extinction coefficient [58].

#### 3.4.2. Absorption Edge Study

At this stage, it is worthwhile to discuss optical absorption, including both the structure and shift of the absorption edge. It is also helpful to have a comprehensive understanding of the potential mechanism of crystalline and noncrystalline materials that are optically induced. Analyses can further verify improvements due to the structures of energy band changes [59]; despite intensive studies on the optical characteristics of polymer composites. [60,61], there is still work to be done on polymer composites based on the Co^2+^-polyphenol metal complex.

The optical absorption spectra help determine and estimate the optical bandgap of the film. The absorption coefficient can be calculated using the following relationship based on the transmittance and reflectance results [54]:

(1) $$\alpha =\frac{1}{t}Ln\left[\sqrt{\frac{{\left(1-R\right)}^{4}+4T^{2}R^{2}-{\left(1-R\right)}^{2}}{2TR^{2}}}\right]$$

where *α* is the absorption coefficient, *T* is the transmittance, and *t* and *R* the thickness and reflectance. To determine *T*, Beer’s law (*T* = 10*^−A^*) can be used. In general, responses to the transmission light from one medium to another include reflection and diffraction, light-transmitting from air to a solid medium, and absorption. As the beam strikes the second medium surface, the responses must be proportional to the summation of the absorption beam *I_A_*, reflected beam intensity *I_R_*, and transmitted beam *I_T_*, as shown in the following relationship:

(2) $$I_{0}=I_{R}+I_{A}+I_{T}$$

The intensity of radiation is designated as W m^−2^. Energy transfer over time and area units indicated the propagation direction at a right angle. As a result of Equation (2), the following relationship is obtained:

(3) $$R+A+T=1_{\;}$$

where *R* represents the reflectivity (*I_R_*/*I_o_*), *A* is the absorptivity (*I_A_*/*I_o_*), and *T* is the transmissivity (*I_T_*/*I_o_*). In other words, *R*, *A*, and *T* refer to reflection, absorption and transmittance, respectively [62]. Thus, reflectance (*R*), as a crucial parameter, can be determined from the refractive index using Equation (3). From an analysis of the optical absorption, the absorption edge is shown to be a decisive parameter in specifying the electronic structure within the material. Optical absorption spectra denote the response of the indirect and direct transition within the bandgap [63].

**Figure 9:**
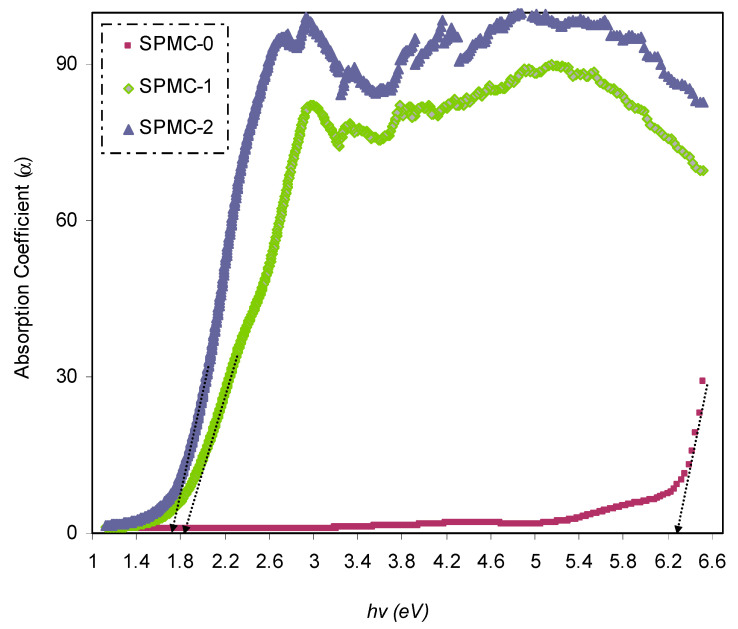
Plot of *α* versus photon energy for the absorption spectra of neat PVA and PVA composites.

Figure 9 shows the absorption coefficient for a variety of photon energies; the absorption edge values are summarized in Table 2. The absorption edge for pure PVA was found to be about 6.3 eV. A broad shift toward a lower photon energy range was observed after incorporating the Co^2+^-polyphenol complex into the system.

A modification of the absorption edge resulted from the dispersion of the complex of Co^2+^-polyphenol within the PVA matrix. In the present study, the value of the absorption edge was in good agreement with that reported previously [62].

Throughout the literature, it has been confirmed that narrowed optical band gaps can be obtained by introducing localized levels inside the energy gap as a consequence of trapping charged species [64]. There is a similarity between this shifting and broadening of the absorption edge to a lower energy range, as in the current results, and those documented for organic and inorganic semiconductors. Organic semiconductors are used extensively in photonic and electronic instruments [65].

#### 3.4.3. Study of the Refractive Index

The refractive index value (*n*) of optical materials is one of the key properties, showing the extent of light-speed changes from one medium to another. For application in optoelectronic instruments, the *n* value of the material of interest must be known. Fundamentally, at constant temperature and pressure, the refractive index (*n*) is dependent upon both density and polarizability of the medium [66]. Therefore, the refractive index of a material is a decisive parameter in determining its optical efficiency. The representation of the complex refractive index of a sample may be summarized as follows:

n*(*λ*) = *n*(*λ*) + *k*(*λ*) (4)

The relationship between *n* and *k* is formulated as follows [54]:

(5) $$n=\left[\frac{\left(1+R\right)}{\left(1-R\right)}\right]+\sqrt{\frac{4\times R}{{\left(1-R\right)}^{2}}}-K^{2}$$

In Equations (4) and (5), *K* is the extinction coefficient equal to *αλ*/4π*t*, and *t* is the film thickness.

Variable *n* can be determined using different techniques. Based on the type of synthesis, two methodologies are widely applied; heavy atoms, such as the polymer matrices, are doped with sulfur and/or halogens [67], and the synthesis of materials of relatively large *n* values from the incorporation of metal or inorganic nanoparticle into polymers [68,69,70]. Two significant challenges are faced in both cases. Firstly, two obstacles exist in the first methodology: the complexity and cost of incorporating heavy atoms into matrices of polymers [71]. Secondly, the aggregation phenomenon occurs with the incorporation of nanofillers comprising inorganic nanoparticles into polymer matrices, for instance, ZrO_2_ (zirconium oxide) [68], TiO_2_ (titanium oxide) [69], and Au (gold) [72]. This leads to the formation of high surface energy and low compatibility with the polymer host. Therefore, in the present research, to change the (*n*) value, a dopant of the Co^2+^-polyphenol complex was inserted into the PVA polymer.

**Figure 10:**
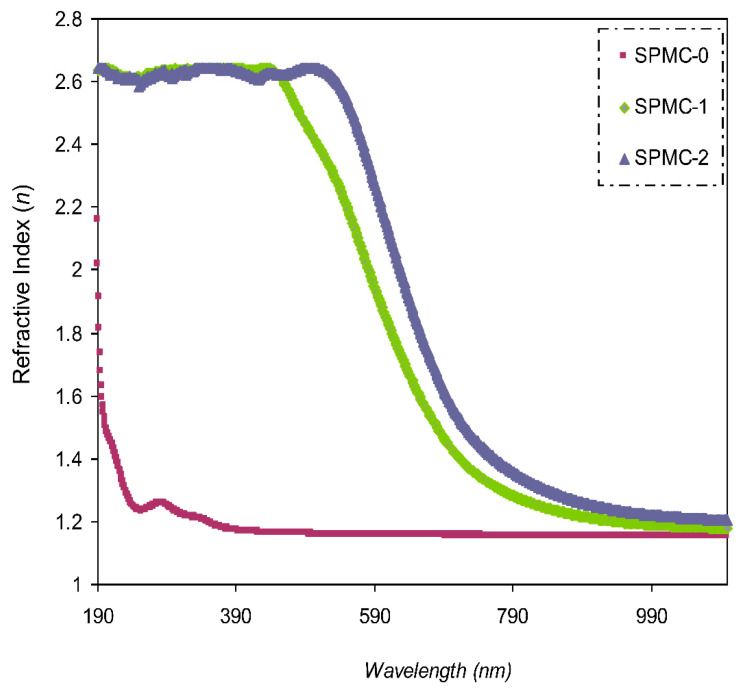
Refractive index spectrum compared to wavelength for the (SPMC-0). (SPMC-1), and (SPMC-2) samples.

The value of (*n*) compared to the wavelength is shown in Figure 10. It was confirmed that larger *n* values can be achieved with incorporated films, indicating considerable dopant dispersion. Figure 10 also shows that as the Co^2+^-polyphenol complex concentration is increased, as is the value of *n*.

Polymers and polymer composites of relatively high *n* are desirable, and have been the focus of many research groups, as their mass, large formability, and flexibility are superior to those of inorganic materials. Many applications of polymers with high (*n*) value have been noted, including in optical storage devices [73], lenses [74], antireflective coatings [75], and optical immersion lithography [76].

#### 3.4.4. Study of the Dielectric Constant

The advantages of polymers, such as their mass, low cost, and minimal dielectric loss, make them ideal for use in energy storage devices. Polymer materials must have a high dielectric constant (*ε_r_*) for use in energy storage applications (i.e., if a large specific capacitance is to be achieved). Composite polymeric materials are among the most reliable methods to modify the *ε_r_* value of polymers; however, several other approaches have been applied. Effectively, nanoparticles can be incorporated into polymers to make polymer nanocomposites. Therefore, polymer nanocomposites (NCs) have been utilized in energy storage capacitors [77,78,79,80] as solid alternatives to battery management systems in microgrids in order to reduce pollution [81,82].

The real and imaginary responses of *ε_r_* are well-established in a relationship in which both values of (*n*) and (*k*) are involved, as shown below [83]:

(6) $$\varepsilon_{r}=n^{2}-k^{2}$$

**Figure 11:**
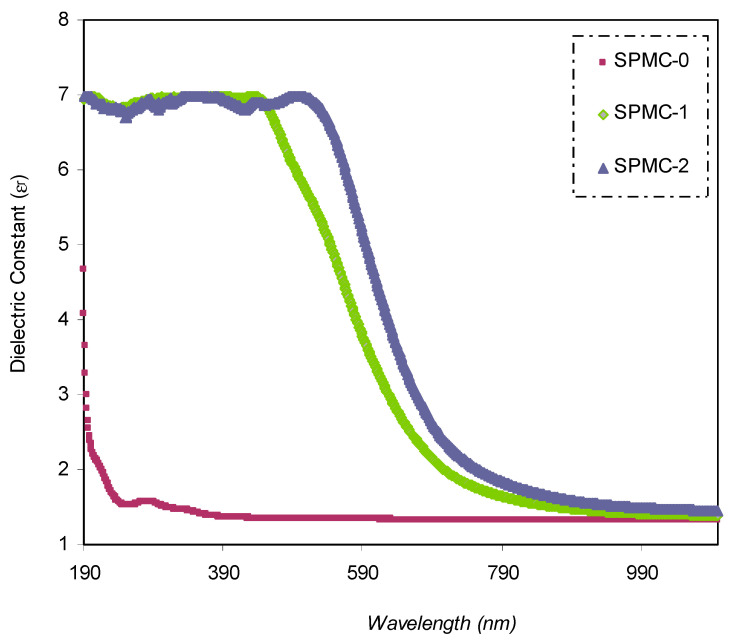
Dielectric constant spectrum against wavelength for the (SPMC-0). (SPMC-1), and (SPMC-2) samples.

The *ε_r_* spectra against wavelength for pure PVA and composite samples are shown in Figure 11. It is seen that as the quantity of Co^2+^-polyphenol complexes increases, *ε_r_* increases as well. This is related to the formation of the density of states within the polymeric film forbidden gap [31].

It is worth noting that modifications in the optical dielectric constant are responsible for the fundamental optical transition in polymer composites. This may be divided into two parts: real, (*ε_r_*) and imaginary, (*ε_i_*). The optical dielectric constant is the measure of the feasibility of losing energy by an electron as it travels through the surface of the bulk material. The real (*ε_r_*) and imaginary (*ε_i_*) parts of the spectra reflect this property. On the one hand, the real part measures the ability of a specific material to attenuate the speed of the electromagnetic wave. On the other hand, the imaginary part shows the energy absorption efficiency due to polarization.

The value of *ε_r_* is obtained from the refractive index (*n*) of the medium ($\varepsilon_{r}=n^{2}-k^{2}$), while *ε_i_* is derived from the extinction coefficient (*k*) ($\varepsilon_{i}=2nk$).

The dielectric response (*ε_∞_*) of the materials at high frequency (short wavelengths) can be determined from the relationship between the wavelength and refractive index, based on the Spitzer–Fan model [84,85]:

(7) $$\varepsilon_{r}=n^{2}-k^{2}=\varepsilon_{\infty }-(\frac{e^{2}}{4\pi^{2}\;C^{2}\;\varepsilon_{o}})\;\times (\frac{N}{m^{*}})\lambda^{2}$$

where *ε_o_* denotes the dielectric constant of free space, *N* is the number of charge carriers, m* is the effective mass (assumed to be 1.16 *m_e_*), and *e*, and *c* have their standard meanings [85,86].

**Figure 12:**
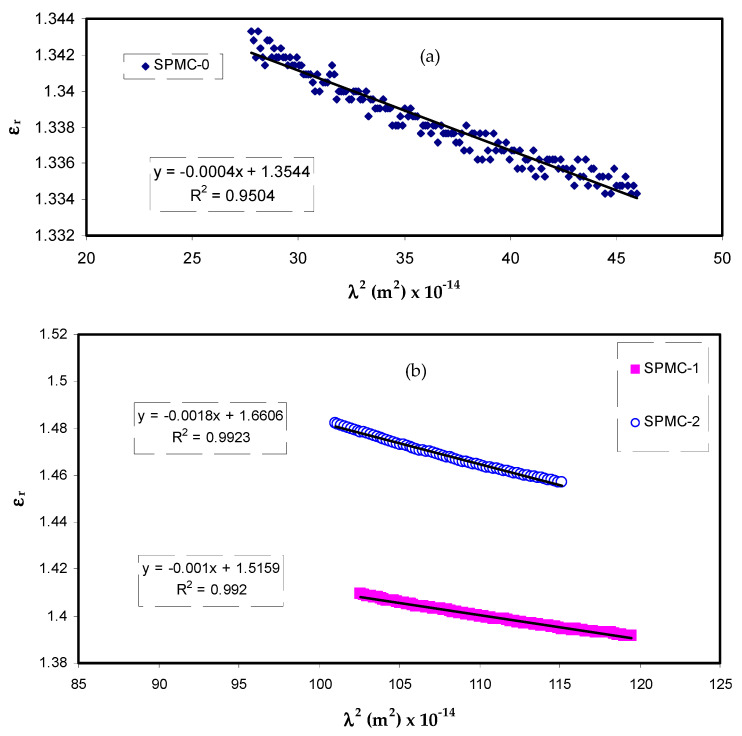
Relationship between *ε_r_* and λ^2^ for (**a**) SPMC_0, (**b**) SPMC_1 and SPMC_2.

The relationship between the values of *ε_r_* and *λ*^2^ in the visible wavelength region is a straight line, as shown in Figure 12. Herein, one can determine the values of *ε_∞_* and N/m* from the slope and intercept of the line in the y axis, respectively, using the parameters presented in Table 3. The values of *ε_∞_*, N/m*, and *N* can be estimated from Equation (7), as summarized in Table 4.

From Table 4, it can be seen that as the filler concentration increases, the values of charge carriers/m* of parent PVA film increase 20 fold, i.e., from 3.68 × 10^55^ to 109 × 10^55^ atoms/m^3^, and the value of *ε_∞_* increases from 1.4 to 3.6. These increasing values of both charge carriers/m* and the *ε_∞_* can be considered indicators of increasing free charge carriers that vigorously participate in the polarization process. In the current study, the values that were estimated for the localized density of states (N/m*) are in good accordance with those reported in the literature using Equation (7) [87,88].

#### 3.4.5. Band Gap Study

Bandgap analyses, i.e., optical band gap energy (*E_g_*), are informative, based on the optical dielectric loss (*ε_i_*). It is necessary to determine the electronic transition behavior using Tauc’s model, as the material’s band structure does not have a significant effect on the optical dielectric function. Indeed, the material band structure and optical dielectric function (ε^*^) exhibit a strong relationship. Thus, the band structures of materials can be clarified from the determination of ε^*^ using UV-vis spectroscopy [31,46,71,89].

The imaginary part of the ε^*^ relies on *n* and *k* values, as shown in the following relationship [27]:

(8) $$\varepsilon_{i}=2nk$$

It is essential to mention that the appearance of the peak in the *ε_i_* spectra is the result of interband transitions [90,91,92]. From the analysis of the *ε_i_* spectra, one can determine the real *E_g_* from the intersection of linear portions of the (*s*) and horizontal axis (*hv*).

In the meantime, complex dielectric function ε* will provide a better understanding of the optical properties of a material. It characterizes the linear reaction of the substance to electromagnetic radiation. The ε* value reflects the essence of the medium in response to electromagnetic wave transmissions. The real transitions between the occupied $\Psi_{k}^{\nu }$ and unoccupied $\Psi_{k}^{c}$ wave functions are represented by the imaginary part *ε*_2_, which is given by [93]:

(9) $$\varepsilon_{2}=\frac{4\pi^{2}e^{2}}{m^{2}\omega^{2}V}\;\sum_{\nu ,\;c,k}^{\;}\left|\langle \Psi_{k}^{\nu }|p_{i}^{\to }{|\Psi }_{k}^{c}\rangle \right|\delta \left(E_{\Psi_{k}^{c}}-E_{\Psi_{k}^{\nu }}-\hbar \omega \right)$$

Equation (9) shows an apparent direct proportionality between *ε*_2_ or *ε_i_* and the band structure $\left(E_{\Psi_{k}^{c}}-E_{\Psi_{k}^{\nu }}\right)$ from the QM perspective. The CDF can be evaluated using simple equations, and intercorrelated to other optical parameters (*n* and extinction coefficient).

**Figure 13:**
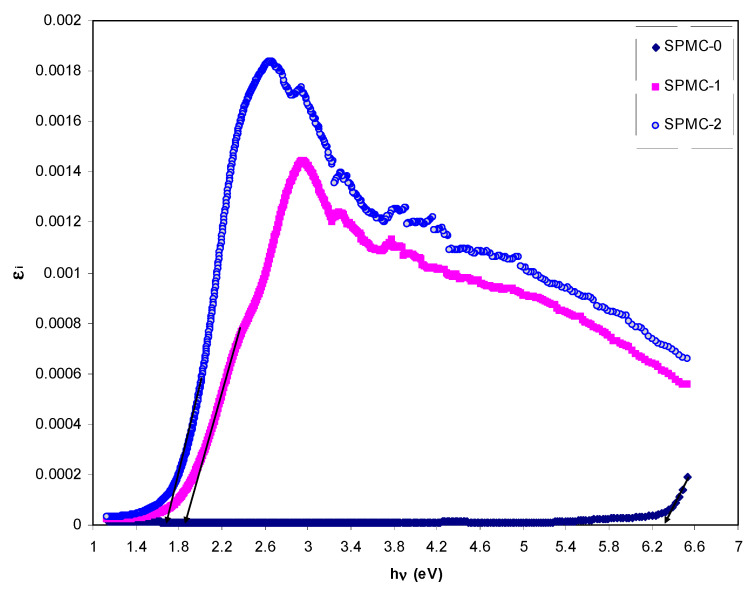
Dielectric loss spectra against photon energy for the (SPMC-0), (SPMC-1), and (SPMC-2) samples.

Figure 13 exhibits the plots of *ε_i_* versus *hν* for parent PVA and composite films. It is seen that there are distinct peaks for all the films. The appearance of the peak in the *ε*_2_ part of the dielectric function can be directly related to interband transitions [94,95]. Therefore, the intercept of linear parts below the peaks on the *hν* axis can be regarded as an accurate bandgap value. The electron transition processes between the bands of a solid are well-defined by the interband absorption process. Furthermore, the absorption edge is caused by the onset of optical transitions through the fundamental bandgaps of a solid material [96]. Yu et al. [97] recently reported that the fundamental absorption edge from dielectric loss against photon energy should be equal, or somewhat similar, to that obtained from Tauc’s relationship.

The optical characteristics of solids can be preliminarily estimated from the CDF, which can be linked to the detectable optical quantities using straightforward equations [88]. Previous studies have emphasized the existence of a strong relationship between the optical dielectric functions (*ε_r_* and *ε_i_*) and the density of localized electronic states within the forbidden gap of the composite films [72,98,99]. Many other decisive parameters, for instance, relaxation time (*τ*), plasma frequency (*ω_p_*) and electrical resistivity (*ρ*), can easily be estimated from the Drude free electron model using the *ε_i_* parameter with the help of (N/m*) values:

(10) $$\varepsilon_{i}=J\;(\frac{1}{\tau })\lambda^{3},\;J=\frac{e^{2}}{8\pi^{3}c^{3}\varepsilon_{o}}\frac{N}{m*}$$

**Figure 14:**
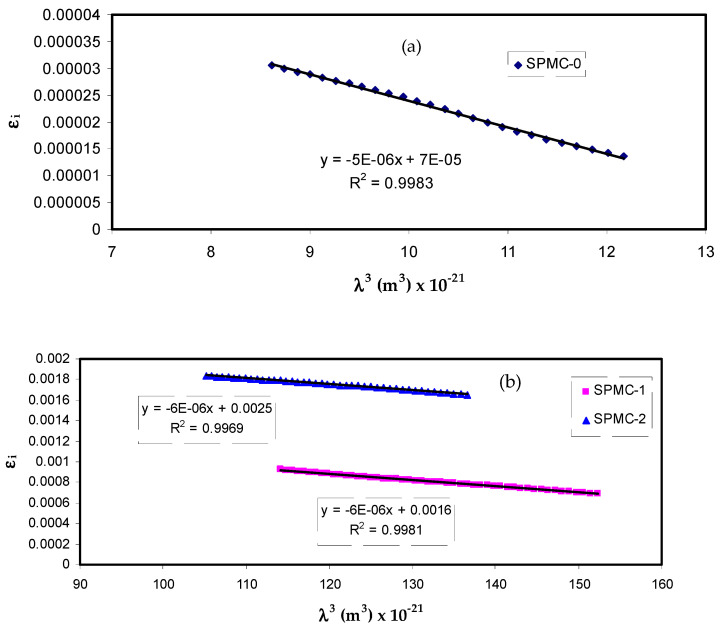
Plots of *ε_i_* versus *λ*^3^ for (**a**) SPMC_0, (**b**) SPMC_1 and SPMC_2.

Figure 14 shows that variation of *ε_i_* corresponds to *λ*^3^ for parent PVA films at different Co^2+^ metal complex quantities in the region where linear behavior is achieved. By inserting the value of N/m* obtained from Equation (10) and the slope of *ε_i_* versus *λ*^3^, the relaxation time (*τ*) values may be calculated. In addition, all other optical properties, such as the optical mobility (*µ_opt_*), electrical resistivity (*ρ_opt_*), and plasma angular frequency of the electron, can be computed from the following relations [100]:

(11) $$\mu_{opt}=\frac{e\tau }{m^{*}}$$

(12) $$\rho_{opt}=\frac{1}{e\mu_{opt}N_{c}}$$

(13) $$w_{p}=\frac{e^{2}N}{\varepsilon_{o}m*}$$

The values of *τ* and *ω_p_* are presented in Table 5. It should also be noted that adding the Co^2+^ metal complex to parent PVA reduces the relaxation time (*τ*), optical mobility (*µ_opt_*), and optical resistivity (*ρ_opt_*), resulting in a faster relaxation response of the nanocomposites to the incident optical electric field in comparison to the unfilled one.

These low *τ*, *µ_opt_* and *ρ_opt_* values are linked to an increase in *n*; in other words, the velocity of light decreases in the medium with a higher refractive index. The addition of the Co^2+^ metal complex also results in an up to 20-fold amplification of the plasma frequency (*ω_p_*) of the electron, i.e., from 0.32 × 10^15^ to 1.77 × 10^15^ Hz. Fortunately, this is in accordance with data documented for polymer nanocomposites and the verified impact of a strong local electric field in the dipole moment of the nanofillers, enhancing the polarization of the material being exposed to an incident electric field [87,98]. This is not the only means by which to estimate the bandgap from optical dielectric loss function; other optical parameters can be determined, as mentioned previously. All these are crucial for choosing suitable materials for optoelectronic applications.

Tauc’s equation can be used to determine the *E_g_* of pure PVA, as well as that of PVA doped with Co^2+^-polyphenol metal complex [25,26]:

(14) $$(\alpha h\upsilon )=B{\left(h\upsilon -E_{g}\right)}^{\gamma }$$

In Equation (14), the *B* value relies on the interband transition probability,(*hv*) denotes the incident photon energy, and (*γ*) is an exponent that specifies the electronic transition type [31].

**Figure 15:**
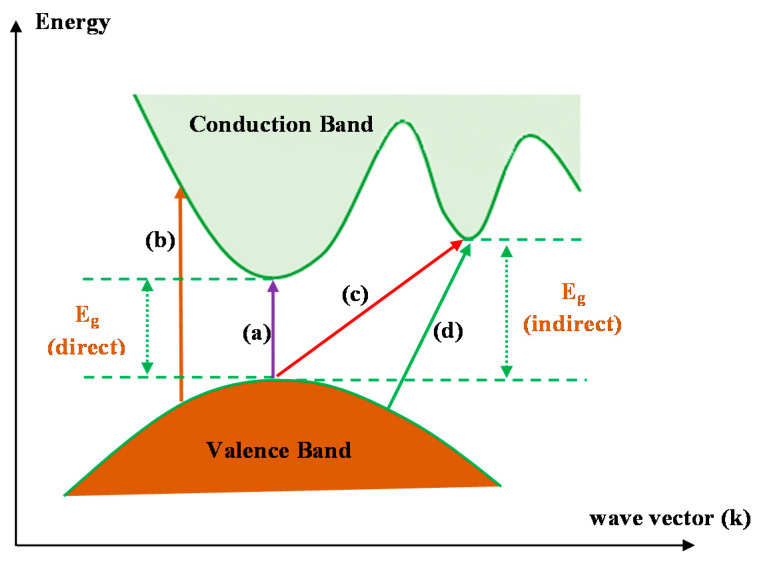
Classes of electronic transition: (**a**) direct allowed, (**b**) direct forbidden, (**c**) indirect allowed, and (**d**) indirect forbidden [101,103].

Figure 15 shows several electronic transitions which occur between the conduction band (CB) and valence band (VB) that are Tauc’s model-dependent [101]. Indeed, when *γ* is 0.5 or 2, direct electron and indirect transitions, respectively, may occur. If *γ* is 1.5 or 3, direct and indirect transitions, respectively, cannot occur [31].

**Figure 16:**
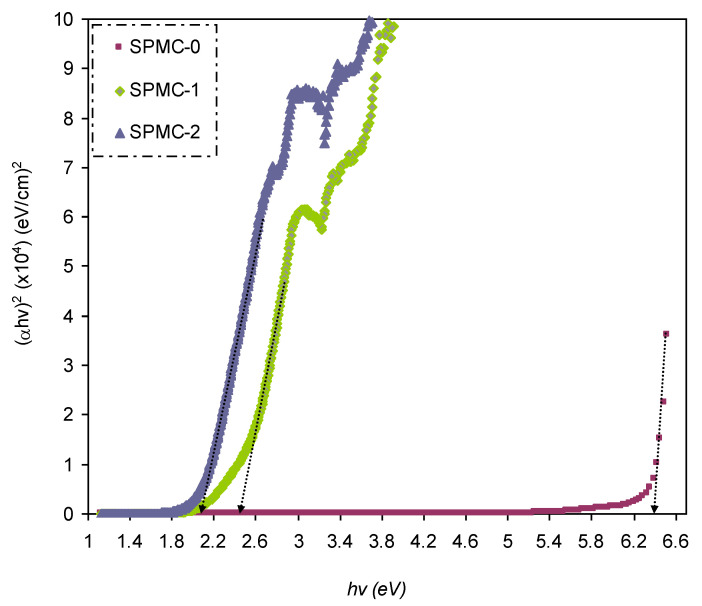
Plot of (*ahv*)^2^ versus photon energy (*hv*) for all films.

**Figure 17:**
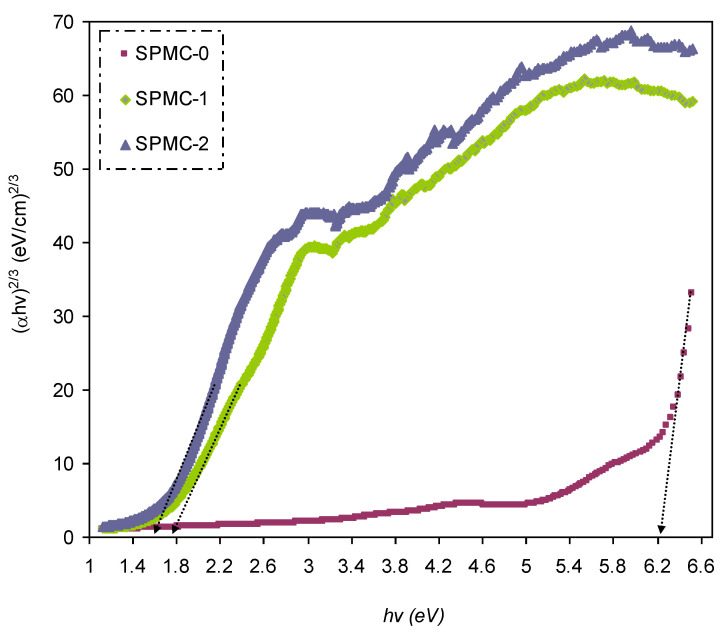
Plot of (*ahv*)^2/3^ versus photon energy (*hv*) for all films.

**Figure 18:**
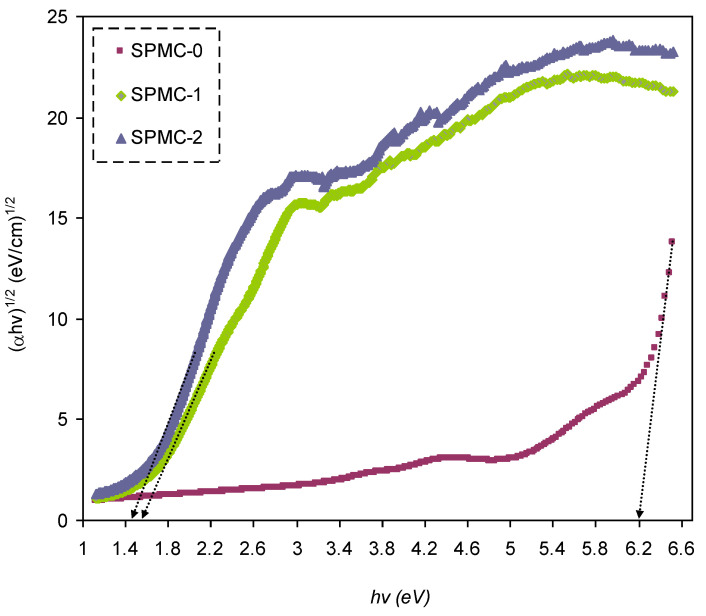
Plot of (*ahv*)^1/2^ versus photon energy (*hv*) for all films.

In Figure 16, Figure 17 and Figure 18, from the extrapolated intersections of the linear part of the (*αhυ*)^1/γ^ plots, in contradiction with *hυ* on the horizontal axis, it is straightforward to determine the value of *E_g_* [31]. It has previously been confirmed that the decrease in *E_g_* results from the introduction of several located states (i.e., trap states) into the forbidden bandgap by the insertion of fillers into the polymer matrix [31,102].

Table 6 presents the values of *E_g_,* which decrease when Co^2+^-polyphenol complex insertion is increased. This may be due to the electronic structure rearrangement of the PVA polymer after addition of Co^2+^-polyphenol complex, leading to imperfections in the PVA polymer [104]. Consequently, the level trapping phenomenon occurs within the bandgap, mediating electron transitions from VB to CB [102]. The two decisive parameters, i.e., *E_g_* and the cut-off energy, can be obtained from Tauc’s model (see Figure 15, Figure 16 and Figure 17) and *ε_i_* (see Figure 13). Based on the values of both parameters, one can determine the electron transition types in the materials [31]. The most likely types of transitions in parent PVA and PVA doped with Co^2+^-polyphenol complex films are direct allowed (*γ* = 1/2) and forbidden (*γ* = 2/3) transitions. The material of choice must have a dominant direct bandgap if it is to be used in light-emitting diodes (LEDs), laser diodes, and photovoltaic cells.

Three materials effectively lower the *E_g_*, i.e., copper nanoparticles, copper powder, and Co^2+^-polyphenol complex. Aziz et al. [60] prepared a polystyrene composite system based on PS-Cu using up to 6 wt.%. copper powder dissolved in PS. It was shown that that the optimum Cu powder is 6 wt.%, in which the *E_g_* value can be decreased from 4.05 eV to around 3.65 eV. Aziz et al. [61] also reported a nanocomposite system based on methylcellulose (MC) as a host polymer and doped with copper (II) sulfide nanoparticles. It was documented that the *E_g_* of MC could be manipulated, dropping from 6.2 eV to 2.3 eV when a quantity of 0.08 M of the (CuS) nanoparticles was used as a dopant.

In the present study, the *E_g_* of PVA was lowered from 6.3 to 1.6 eV when 60 mL Co^2+^-polyphenol complex was added. Interestingly, it was shown that the Co^2+^-polyphenol complex was effective at lowering the *E_g_* and superior to both copper nanoparticles and powder incorporations. Furthermore, bandgap manipulation was made possible due to the incorporation of the cobalt complex, giving rise to a low value of *E_g_*, and an environmentally sustainable approach for the synthesis of polymer composites.

The refractive index dispersion of the prepared films was analyzed using experimental data fitting based upon the Wemple–DiDomenico (WDD) single oscillator model. The refractive index and its dispersion behavior are among the key optical material properties to be studied. The refractive index dispersion is crucial in evaluating the optical communication for spectral dispersion [105].

In the normal region, the single oscillator model presented by WD can be implemented to examine the refractive index dispersion [106,107]. This is carried out by introducing a dispersion energy parameter (*E_d_*) to gauge the force of interband optical transition. This parameter combines both the coordination number and the charge allocation in each unit cell, and correlates with chemical bonding [108,109]. However, a single oscillator parameter (*E_o_*) is directly proportional to the oscillator energy (i.e., the average energy bandgap). The refractive index and the photon energy below the interband absorption edge may be related, as shown in the following semiempirical equation:

(15) $${\left(n^{2}-1\right)}^{-1}=\frac{E_{o}}{E_{d}}-\left(\frac{1}{E_{o}E_{d}}\right){\left(h\gamma \right)}^{2}$$

where *E_o_* and *E_d_* are constants denoting the single-oscillator and dispersive energies, respectively. Parameters *E_o_* and *E_d_* relate to the average excitation energy and structure disorder, enhancing the optical transition within the material’s band structure.

**Figure 19:**
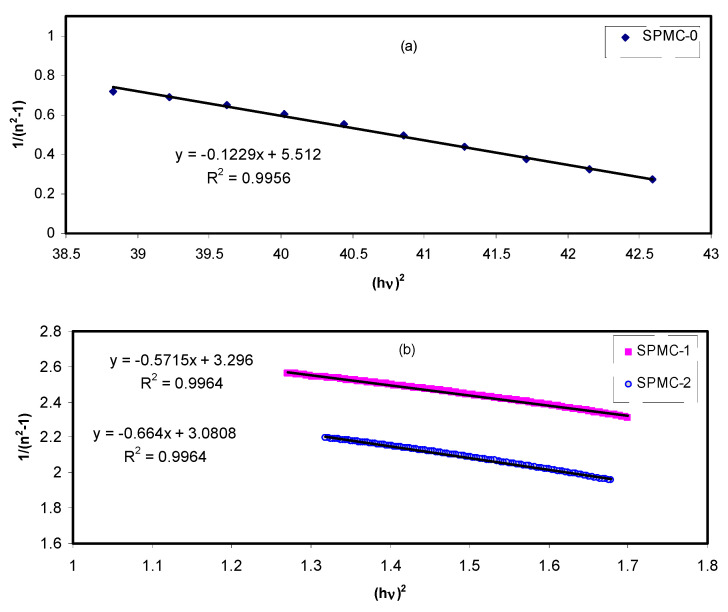
Variation of (n^2^ − 1)^1/2^ versus photon energy (*hv*)^2^ for the (**a**) SPMC_0, (**b**) SPMC_1, and SPMC_2 samples.

The $\left(\frac{1}{E_{o}E_{d}}\right)$ values are obtained from the slope of the linear portion of ${\left(n^{2}-1\right)}^{-1}$ verses ${\left(h\gamma \right)}^{2}$. The $\frac{E_{o}}{E_{d}}$ values are determined from the intersection of the graph with the y-axis, as shown in Figure 19.

The values of *E_o_*, *E_d_*, and parameters determined from the Wemple-Didomenic model are summarized in Table 7.

## 4. Conclusions

In conclusion, it seems that the black tea leaf extract can be used successfully in the preparation of a polymer composite of Cd^2+^-polyphenol complex after synthesis of the PVA-Co^2+^-polyphenol composite. The fundamental characteristics of the polymer composite have been characterized, including the type of transitions and gap energy (*E_g_*), absorption edge, refractive index (*n*), dielectric constant (*ε_r_*), and dielectric loss (*ε_i_*). The optical bandgap could be determined using optical dielectric loss, whereas the types of electronic transitions could be estimated by applying Tauc’s model. Thus, the optical dielectric loss has been precisely analyzed. The composite of the PVA-Co^2+^-polyphenol complex was shown to alter *E_g_*, i.e., a relatively low value was obtained. A direct bandgap enhancement of the polymer composite was observed in the amorphous structure. The XRD outcome indicated a decline in peak intensity of neat PVA after the incorporation of Co^2+^-polyphenol complex. The change in the intensity of the FTIR bands in the PVA after the addition of the Co^2+^-polyphenol complex can be considered strong evidence of the extent of interaction among the polymer composite components. The easy and green nature of the methodology is significant, with potential for large-scale applications in electronic devices. This composite may enhance the performance of electrochemical energy storage devices.

## Figures and Tables

**Table 1:** FTIR results of PVA and doped PVA with Co^2+^-metal complex.

Attributions	Wavenumber (cm^−^^1^)		
	SPMC_0	SPMC_1	SPMC_2
O-H stretching	3310	3308	3305
C–H stretching	2908	2915	2916
C=O stretching	1640	1648	1647
C=C stretching	1623	1624	1630
CH_2_ Wagging	1410	1418	1423
C-O-H bands	1150	1158	1162
C–H rocking	824	826	828

**Table 2:** Absorption edge values for the (SPMC-0). (SPMC-1), and (SPMC-2) samples.

Films	Absorption Edge (eV)
SPMC-0	6.3
SPMC-1	1.83
SPMC-2	1.75

**Table 3:** A variety of physical parameters used for the calculation of localized density of states (N/m*) for PVA containing Co^2+^ metal complex.

Physical Parameters	Values
*m_e_*	9.109 × 10^−31^ Kg
*e*	1.602 × 10^−19^ coulombs
*ɛ_o_*	8.85 × 10^−12^ F/m
π	3.14
*c*	2.99 × 10^8^ m/s
m*	10.566 × 10^−31^ Kg

**Table 4:** The localized density of states (N/m*) and optical dielectric constant for PVA containing Co^2+^ metal complex.

Film Code	N/m* × 10^55^ (m^3^/kg)	*ɛ_∞_*	*ω_p_* × 10^14^
SPMC-0	4.91	1.35	3.76
SPMC-1	12.3	1.51	5.95
SPMC-2	22.1	1.66	7.99

**Table 5:** Variations of *τ* and *ω_p_*, (*µ_opt_*), and (*ρ_opt_*), obtained from the slope of *ε_i_* versus *λ*^3^.

Film Code	N/m*	*τ*	*μ*(*opt*)	*ρ*(*opt*)
SPMC-0	4.91 × 10^+55^	4.25 × 10^−15^	6.41 × 10^−4^	1.88 × 10^−4^
SPMC-1	1.23 × 10^+56^	8.85 × 10^−15^	13.35 × 10^−4^	3.61 × 10^−5^
SPMC-2	2.21 × 10^+56^	1.59 × 10^−14^	24.04 × 10^−4^	1.12 × 10^−5^

**Table 6:** Presents the *E_g_* values from Tauc’s method and *ε_i_* plot.

Sample Code	γ = 2/3	γ = 2	γ = 1/2	Dielectric Loss
SPMC_0	6.22	6.4	6.2	6.35
SPMC_1	1.76	2.45	1.6	1.85
SPMC_2	1.6	2.1	1.5	1.65

**Table 7:** Optical bandgap from the theoretical Wemple-DiDomenico single oscillator model.

Sample Code	*E_d_*	*E_o_*
SPMC-0	1.214707	6.693037
SPMC-1	0.728961	2.39828
SPMC-2	0.699263	2.15373

## Data Availability

Exclude this statement if the study did not report any data.
